# Identification, Functional Study, and Promoter Analysis of *HbMFT1*, a Homolog of *MFT* from Rubber Tree (*Hevea* *brasiliensis*)

**DOI:** 10.3390/ijms17030247

**Published:** 2016-03-02

**Authors:** Zhenghong Bi, Xiang Li, Huasun Huang, Yuwei Hua

**Affiliations:** 1 College of Agronomy, Hainan University, Haikou 570228, China; bizhenghong9999999@163.com; 2 Key Laboratory of Rubber Biology of the Ministry of Agriculture, Rubber Research Institute, Chinese Academy of Tropical Agricultural Sciences, Danzhou 571737, China; 3 College of Environment and Plant Protection, Hainan University, Haikou 570228, China; lixiaoxiao1986@hotmail.com

**Keywords:** Phosphatidyl ethanolamine-binding protein (PEBP) family, *MFT* homolog, *Arabidopsis*, Rubber tree, Germination, Flowering

## Abstract

A homolog of *MOTHER OF FT AND TFL1* (*MFT*) was isolated from *Hevea* *brasiliensis* and its biological function was investigated. Protein multiple sequence alignment and phylogenetic analysis revealed that *HbMFT1* conserved critical amino acid residues to distinguish MFT, FLOWERING LOCUS T (FT) and TERMINAL FLOWER1 (TFL1)-like proteins and showed a closer genetic relationship to the *MFT*-like group. The accumulation of *HbMFT1* was generally detected in various tissues except pericarps, with the highest expression in embryos and relatively higher expression in roots and stems of seedlings, flowering inflorescences, and male and female flowers. *HbMFT1* putative promoter analysis showed that tissue-specific, environmental change responsive and hormone-signaling responsive elements were generally present. *HbMFT1* was strongly induced under a short-day condition at 28 °C, with the highest expression after the onset of a day. Overexpression of *HbMFT1* inhibited seed germination, seedling growth, and flowering in transgenic *Arabidopsis*. The qRT-PCR further confirmed that *APETALA1* (*AP1*) and *FRUITFULL* (*FUL*) were drastically down-regulated in 35S::*HbMFT1* plants. A histochemical β-glucuronidase (GUS) assay showed that HbMFT1::GUS activity was mainly detected in stamens and mature seeds coinciding with its original expression and notably induced in rosette leaves and seedlings of transgenic *Arabidopsis* by exogenous abscisic acid (ABA) due to the presence of ABA *cis*-elements in *HbMFT1* promoter. These results suggested that *HbMFT1* was mainly involved in maintenance of seed maturation and stamen development, but negatively controlled germination, growth and development of seedlings and flowering. In addition, the *HbMFT1* promoter can be utilized in controlling transgene expression in stamens and seeds of rubber tree or other plant species.

## 1. Introduction

Rubber tree, a member of *Euphorbiaceae* family [1], is a monoecious species with male and female flowers on the same inflorescence and is known as an economically important crop in that it can produce natural rubber which is widely used in various aspects, such as in the rubber industry, medical health and items used in daily life because of its particular features of strong flexibility, good insulation, and plasticity as well asits waterproof quality. For higher yield many of the agronomic and economic traits of rubber trees need to be improved, such as low temperature tolerance, strong-wind resistance, as well as pest and disease resistance. Conventional breeding programs have been conducted to improve these traits for many decades, and it has taken more than 28 years to breed and select a new clone for commercial production. Rubber trees have a life span of more than 30–35 years, and an immaturity of five to eight years [1,2,3]. Therefore, molecular breeding provides an advantageous genetic improvement method to obtain desirable traits and speed up the *Hevea* breeding program. Simultaneously, tissue-specific promoters used for control of incorporation and expression of exogenous genes will contribute greatly to improvement of the traits in special organs and tissues.

The phosphatidyl ethanolamine-binding protein (PEBP) family is present in various organisms such as archaea, prokaryotes and eukaryotes [4,5,6], and has been shown to have conservative functions during evolution [7,8]. In animals, the PEBP family functioned as Raf kinase inhibitors, thereby regulating cell growth [9,10]. Moreover, the plant PEBP family played an important role in control of plant development and architecture. Typically, the *AtPEBP* family, which contains six members, was earlier identified and investigated in the model plant *Arabidopsis*. FLOWERING LOCUS T (FT) and *FT* mRNA were confirmed to be long-distance mobile signals translocating from vascular tissue over a long distance to shoot apical meristem, and then FT protein combined with FLOWERING LOCUS D (FD), a bZIP transcription factor, triggered flower initiation [11,12,13,14,15]. *TWIN SISTER OF FT* (*TSF*) functioned redundantly with *FT* in regulating floral initiation, and both *TSF* and *FT* were regulated by CONSTANS (CO) protein, which was post-transcriptionally activated when the *CO* transcript was expressed in long-day condition [16,17]. *TERMINAL FLOWER1* (*TFL1*) was identified as a flowering inhibitor although it showed relative high similarity (about 59%) with FT. *TFL1* was restricted in inner cells of shoot apical meristem (SAM), but it could move to lateral regions interacting with FD to inhibit the accumulation of floral meristem identity genes, such as *LEAFY* (*LFY*) and *APETALA1* (*AP1*) [18]. *BROTHER OF FT AND TFL1* (*BFT*) was reported to act similarly to *TFL1*, inhibiting flowering. However, overexpression of *BFT* could not rescue the terminal flower phenotype of the *tfl1* mutant but negatively regulates formation of axillary inflorescences [19]. *ARABIDOPSIS THALIANA CENTRORADIALIS* (*ATC*) in *Arabidopsis* was induced in short-day condition. *ATC* also interacted with FD as antiflorigen to inhibit floral initiation after moving from vasculature to the apex [20]. *MOTHER OF FT AND TFL1* (*MFT*) may be ambiguous in function. Yoo *et al.* [21] revealed that *MFT* weakly accelerated flower formation, acting similarly to *FT* to some degrees, but no notable differences between the wild-type plants and the *mft-1* mutants. Furthermore, Xi *et al.* [22] confirmed that *MFT* was specifically induced in embryo and acted as a negative regulator, resisting the suppressor effect of ABA on germination. In other plant species, *MFT* homologs have been predominantly detected in seeds or embryos, such as *gymnosperm* *Picea* *abies* [23], *Populus* *nigra* [24], and wheat [25]. However, *MFT*-like genes were predominantly detected in gametangia and sporophytes in *Physcomitrella patens* [26].

As of this writing, there has been no report about the function of *MFT* homologs in rubber tree. In this study, we cloned and identified the *HbMFT1* gene. Ectopic overexpression of *HbMFT1* inhibited seed germination, aerial part and root growth, and delayed floral initiation. HbMFT1::GUS fusion activity mainly existed in stamens and mature seeds of transgenic *Arabidopsis* plants, coinciding with its expression in rubber tree. Therefore, *HbMFT1* may be a multifunctional regulator and function in distinct aspects of development, mainly involved in maintenance of seed maturation and development of stamens. And the *HbMFT1* promoter may be a candidate for driving target genes preferential expression in seeds and stamens.

## 2. Results

### 2.1. Isolation and Phylogenetic Analysis of HbMFT1 from Rubber Tree

**Figure 1:**
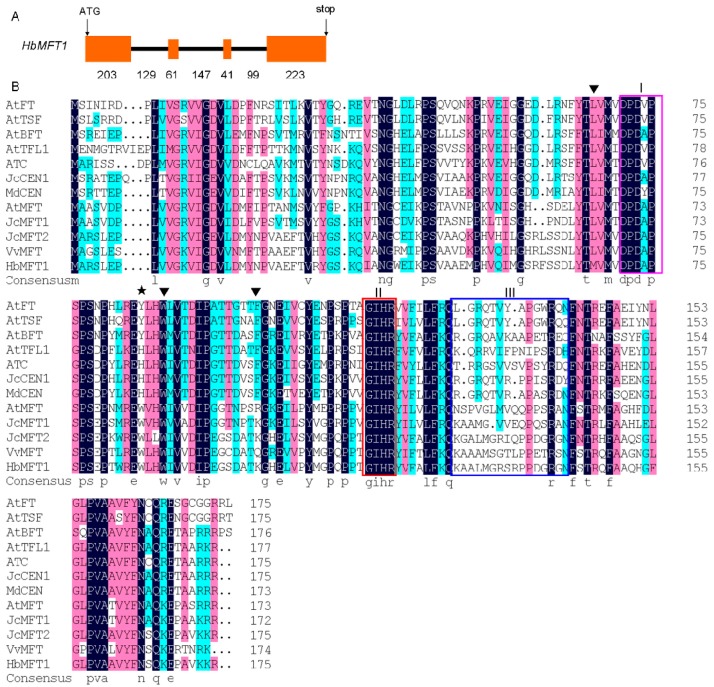
(**A**) Genomic organization of *HbMFT1*. Yellow boxes represent exons. Lines represent introns; (**B**) Protein multiple alignment between deduced amino acid sequence of HbMFT1 in rubber tree and phosphatidyl ethanolamine-binding protein (PEBP) family of other species. Sequence alignment was carried out using DNAMAN 6.0 software (http://www.lynnon.com/). Three triangles refer to the intron positions. I, D-P-D-x-P motif. II, G-x-H-R motif. III, the region is essential for FT/TFL1-like activity in exon IV. An asterisk indicates amino acids that are related to antagonistic functions between TFL1 and FT protein. Different colors refer to the different homology levels of aligned amino acid residues among MFT homologs. Darkblue represents 100% identity. Hotpink represents more than 75% identity. Turquoise represents more than 50% identity. The aforementioned proteins and their accession numbers: *Arabidopsis* (AtMFT, NP_173250.1; AtFT, NP_176726.1; AtTSF, NP_193770.1; AtBFT, NP_201010.1; AtTFL1, NP_196004.1; ATC, NP_180324.1), *Jatropha curcas* (JcCEN, NP_001295672.1; JcMFT1, KC874668; JcMFT2, KF944352), *Malus domestica* (MdCEN, NP_001280770.1), *Vitis vinifera* (VvMFT, NP_001267935.1).

**Figure 2:**
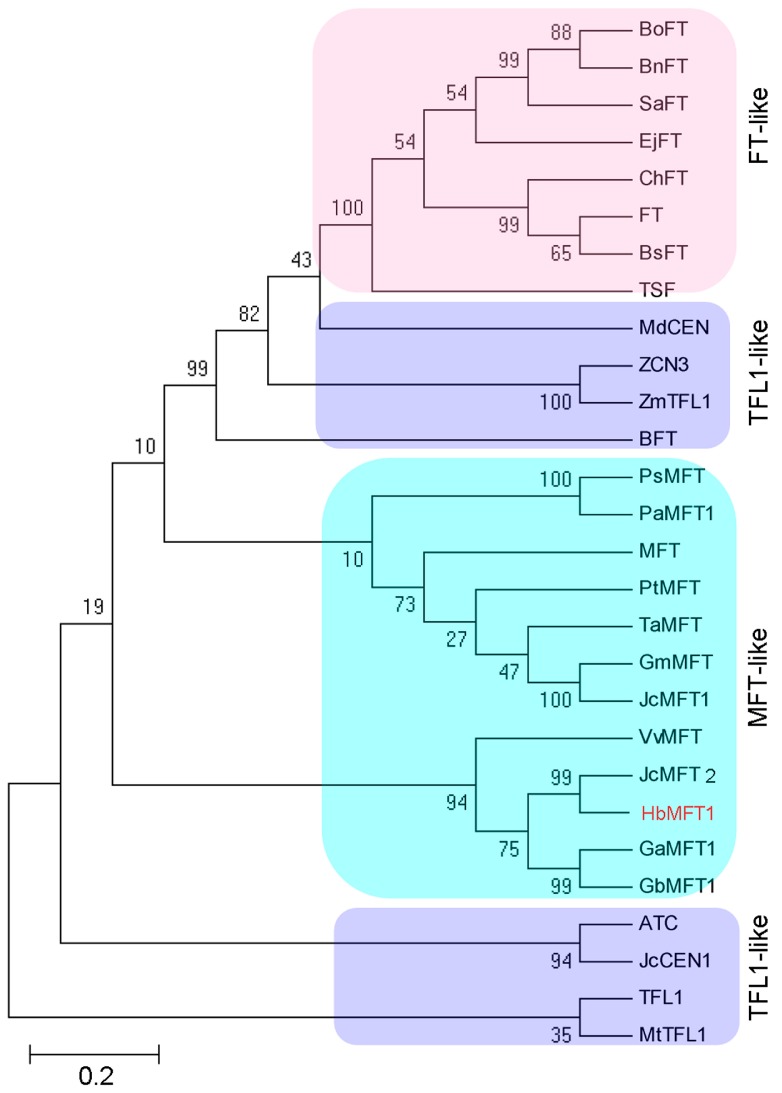
Phylogenetic analysis of the members in PEBP family. The tree was constructed using the Neighbor-Joining (N-J) method for members of the PEBP family in *Hevea brasiliensis* (HbMFT1), *Jatropha curcas* (JcCEN1, NP_001295672.1; JcMFT1, KC874668; JcMFT2, KF944352), *Arabidopsis thaliana* (TFL1, NP_196004.1; TSF, NP_193770.1; FT, NP_176726.1; MFT, NP_173250.1; BFT, NP_201010.1; ATC, NP_180324.1), *Triticum aestivum* (TaMFT, BAK78908.1), *Populus trichocarpa* (PtMFT, XP_002321507.1), *Glycine max* (GmMFT, ACA24491.1), *Picea abies* (PaMFT1, AEH59565.1), *Pinus sylvestris* (PsMFT, AIJ02001.1), *Gossypium barbadense* (GbMFT1, AGJ98454.1), *Gossypium arboreum* (GaMFT1, KHG10593.1), *Sinapis alba* (SaFT, ACM69283.1), *Brassica napus* (BnFT, ACY03404.1), *Brassica oleracea* (BoFT, ACH86033.1), *Eutrema japonicum* (EjFT, ADV18466.1), *Boechera stricta* (BsFT, AIU56794.1), *Cardamine hirsuta* (ChFT, AKC05615.1), *Zea mays* (ZmTFL1, ABI98712.1; ZCN3, ABX11005.1), *Medicago truncatula* (MtTFL1, XP_013443336.1), *Vitis vinifera* (VvMFT, NP_001267935.1), *Malus domestica* (MdCEN, NP_001280770.1). All of the protein sequences were downloaded from the NCBI according to their accession numbers. Abbreviations: ATC, ARABIDOPSIS THALIANA CENTRORADIALIS; BFT, BROTHER OF FT AND TFL1; FT, FLOWERING LOCUS T; MFT, MOTHER OF FT AND TFL1; TFL1, TERMINAL FLOWER1; TSF, TWIN SISTER OF FT; CEN, CENTRORADIALIS.

According to the genome database, we obtained two putative *MFT* homologs through the Basic Local Alignment Search Tool (BLAST) program, designated as *HbMFT1* (accession number: KU365051) and *HbMFT2*, of which only *HbMFT1* mRNA (accession number: KU365050) accumulation could be detected in the leaf transcriptome. However, the *HbMFT2* cDNA fragment was also not successfully cloned from sampled tissues other than leaves even though several pairs of specific primers were designed, suggesting that the function of *HbMFT2* may be degenerated during evolution. A 2267 bp 5′ flanking region upstream of *HbMFT1* coding region was cloned using a pair of specific primers and a 151 bp 3′ untranslated region (UTR) was obtained by 3′ rapid amplification of cDNA ends (RACE). Through sequence analysis, *HbMFT1*, which encoded 175 aa, conserved the characteristic genomic organization of the *PEBP* gene family, including four exons and three introns (Figure 1A). Protein multiple sequence alignment analysis revealed that HbMFT1 possessed the characteristics of MFT. At position His88/Tyr85 in the TFL1/FT-like protein, HbMFT1 contained other Trp88-like MFT homologs, suggesting that Trp88 is highly conserved for function in MFT homologs among different species because His88/Tyr85 conferred TFL1/FT function in other plants [27,28]. HbMFT1 protein was more similar to JcMFT2, VvMFT, JcMFT1, AtMFT (showed 88.29%, 72.57%, 58.29% and 58.29% sequence identity, respectively) than to FT and TFL1 (showed 44.38% and 46.93% identity, respectively). Like other members of the PEBP family, MFT-like proteins contained intact D-P-D-x-P and G-x-H-R motifs (Figure 1B), both of which were in favour of the combination among the ligand-binding sites [7]. Phylogenetic analysis showed that HbMFT1 was most closely related to JcMFT2, GaMFT1, GbMFT1 and VvMFT*,* and was clustered into MFT-like groups (Figure 2). Interestingly, the TFL1-like proteins were divided into two distinct subgroups, in which BFT, MdCEN, ZCN3 and ZmTFL1 showed more similarity to FT-like proteins. However, all of the TFL1-like proteins in the phylogenetic tree contained two conservative amino acid residues such as TFL1 at His88 and Asp144 with an exception for BFT, which contained Tyr 85 and Glu141. It has been speculated that the presence of a charged hydrogen bond between Asp144 and His88 in TFL1 determined TFL1 activity [19]. In *Arabidopsis*, BFT has been confirmed to function redundantly with TFL1 when overexpressed in *Arabidopsis* because of the presence of a hydrogen bond between Tyr 85 and Glu141 like TFL1 [19]. Therefore, MdCEN, ZCN3 and ZmTFL1 also function similarly to TFL1 rather than FT, and fall into a TFL1-like subgroup together with BFT.

### 2.2. HbMFT1 Expression Analysis in Rubber Tree

**Figure 3:**
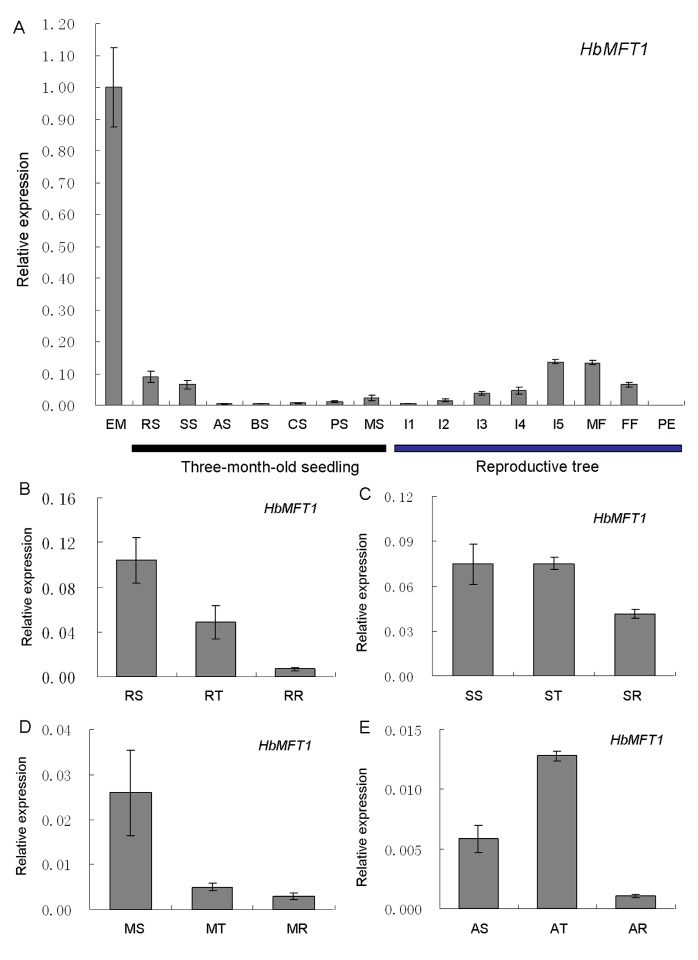
Expression analysis of *HbMFT1* in rubber tree. (**A**) Tissue-specific expression analysis of *HbMFT1*. EM, RS, SS, AS, BS, CS, PS, MS, embryos and roots, stems, shoot apices, bronze, color change, pale-green and mature leaves of three-month old seedlings; PE, MF, FF, pericarps, open male and female flowers; I1, I2, I3, I4 and I5, five different developmental-stage inflorescences; (**B**–**E**) expression of *HbMFT1* in roots, stems, mature leaves and shoot apices of rubber trees at different ages (three months, two years and ten years). RS, SS, MS and AS, roots, stems, mature leaves and shoot apices of the three-month-old seedlings; RT, ST, MT and AT, roots, stems, mature leaves and shoot apices of the two-year-old trees; RR, SR, MR and AR, roots, stems, mature leaves and shoot apices of the ten-year-old trees (adult trees). *HbRH2b*, *HbRH8* and *HbYLS8* were used as reference genes for qRT-PCR analysis. Values were means ± SE from three independent biological replicates.

The tissue-specific expression analysis was conducted by qRT-PCR and the result showed that *HbMFT1* could be detected in various tissues except pericarp, with the highest expression in embryo. *HbMFT1* also showed stronger transcript accumulation in roots and stems of three-month old seedlings, and flowering inflorescences (namely I5), male and female flowers of mature rubber trees (Figure 3A). It was worthwhile to note that *HbMFT1* was gradually increased along with development of leaves and inflorescences (Figure 3A). We also found that its expressions in roots, stems and leaves were progressively decreased from the three-month-old seedlings to the ten-year-old rubber trees (Figure 3B–D). However, the expression in shoot apices was increased from the three-month-old seedlings to the two-year-old trees, but was decreased sharply in the ten-year-old rubber trees, significantly lower than that of the three-month-old seedlings (Figure 3E).

### 2.3. Cloning and cis-Elements Analysis of the HbMFT1 Promoter

Based on genome database (data unpublished), a 2267 bp putative promoter fragment upstream of *HbMFT1* was obtained from cultivated rubber tree 7-33-97 and putative *cis*-acting elements were analyzed with the PLACE database (http://www.dna.affrc.go.jp/htdocs/PLACE/) [29]. Various putative plant regulatory elements in the *HbMFT1* promoter are shown in Table S1. There were some tissue-specific elements such as GTGANTG10 and POLLEN1LELAT52 for pollen expression, TAAAGSTKST1 for guard cell expression, EBOXBNNAPA, MYCATERD1, MYCATRD22 and 2SSEEDPROTBANAPA for storage-protein expression, CANBNNAPA and PROXBBNNAPA for embryo- and endosperm-specific expression, RHERPATEXPA7 for root hair-specific expression, RAV1AAT and RAV1BAT for rosette leaf and root expression, NODCON2GM and NODCON1GM for nodule expression, DOFCOREZM and AACACOREOSGLUB1 for endosperm expression, GATABOX, SEF4MOTIFGM7S, DPBFCOREDCDC3 and SEF3MOTIFGM for seed or embryo expression, SITEIIATCYTC for anther- and meristem-specific expression, and TGTCACACMCUCUMISIN for fruit expression. In addition, there were other putative regulatory elements in response to environmental cues and hormone signals, such as temperature responsive elements, water stress responsive elements, light-responsive elements, disease responsive elements, circadian-regulated elements and the elements in response to hormones including salicylic acid, gibberellin acid (GA), jasmonic acid (JA), abscisic acid (ABA) and auxin. Therefore, it is tempting to speculate that the activity of the *HbMFT1* promoter is limited in specific tissues because of the presence of tissue-specific *cis*-acting elements and may be regulated by various environmental stresses and hormone signals.

### 2.4. HbMFT1 Promoter Activity Analysis under Different Photoperiods and Temperatures

**Figure 4:**
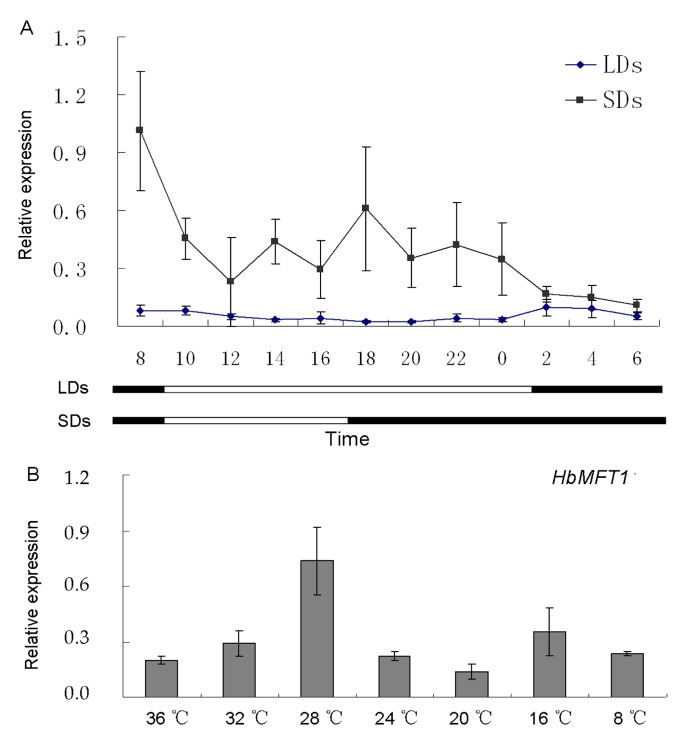
Expression changes of *HbMFT1* in response to different photoperiods and temperatures. (**A**) Expression profiles of *HbMFT1* in long-day (LD) and short-day conditions (SD); (**B**) Expression changes of *HbMFT1* at different temperatures. Results were from three independent biological replicates.

Given that many *cis*-acting elements responsive to light are present in the *HbMFT1* promoter, we carried out the expression analysis under long-day and short-day conditions, respectively, in mature leaves, in which the expression was higher than that of the other development-stage leaves (Figure 3A). The qRT-PCR results revealed that the *HbMFT1* transcript was mainly induced under the short-day conditions, came to a peak after the onset of day, and then progressively decreased till the fourth hour. Small oscillations were subsequently generated and lasted for the remaining four hours of the light period and the previous ten hours of darkness (Figure 4A). However, extremely weak expression of *HbMFT1* was observed under the whole long-day condition. Similarly to the expression in short-day conditions, *HbMFT1* also showed slight oscillations after entering darkness, lasting six hours (Figure 4A). In addition, the abundance of the *HbMFT1* transcript was modulated by temperature. As shown in Figure 4B, as the temperature decreased from 36 to 28 °C, *HbMFT1* transcript accumulation was gradually increased, and then progressively reduced as the temperature continued to decrease to 20 °C, at which minimal expression level was detected. However, small fluctuations occurred at 16 and 8 °C. This result indicated that 28 °C is the best inducing temperature for *HbMFT1* mRNA accumulation.

### 2.5. Characterization of HbMFT1::GUS (β-Glucuronidase) Fusion in Transgenic Arabidopsis

**Figure 5:**
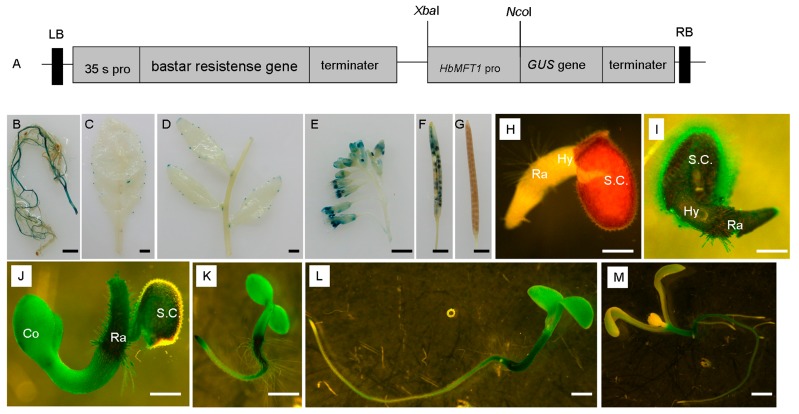
HbMFT1::GUS (β-glucuronidase) activity analysis in transgenic *Arabidopsis* plants. (**A**) Schematic of T-DNA structure of pCAMBIA3301 recombinant construct. Various tissues of adult transgenic plant harboring HbMFT1::GUS fusion, including root (**B**); rosette leaf (**C**); stem with cauline leaves and axillary meristems (**D**); flowering inflorescence (**E**); mature silique (**F**); wild-type mature silique (**G**); wild-type seedling at 12 h after germination (HAG) (**H**); seedling at 12 HAG (**I**); seedling at 1 day after germination (DAG) (**J**); 3 DAG (**K**); 5 DAG (**L**); 7 DAG (**M**). bar = 2 mm for (**B**–**G**), bar = 0.2 mm for (**H**–**J**), bar = 1 mm for (**K**–**M**). Co: cotyledon, Ra: radicle, Hy: hypocotyl, S.C.: seed coat.

A binary vector containing *HbMFT1::GUS* fusion fragment was constructed and introduced into *Arabidopsis* (Figure 5A). Two independent lines were obtained in the T1 generation, of which the roots, stems with caulines and axillary meristems, rosette leaves, flowering inflorescences and mature siliques were used for histochemical GUS staining analysis 30 days after germination. The result showed that both two lines exhibited similar GUS expression profiles, and that GUS activity was strongly observed in roots (Figure 5B), the base of axillary meristems (Figure 5D), stamens of flowers (Figure 5E) and mature seeds located in mature siliques (Figure 5F) but weakly detected in the tips and sides of cauline and rosette leaves (Figure 5C,D), sepals and petals (Figure 5E). However, no GUS activity was detected in stems (Figure 5D). We further examined HbMFT1::GUS activity at 12 h after germination (HAG), and 1, 3, 5 and 7 days after germination (DAG) in T2 generation. Strong GUS activity appeared in whole seedlings and seed coats, with the highest expression in radicles from 12 HAG to 1 DAG (Figure 5I,J), after which the GUS activity was decreased gradually in cotyledons (Figure 5K–M). At three DAG, root meristems were elongated and the strongest GUS activity was still located in radicles also in root tips whereas faint activity was detected in the newly elongated regions of the roots (Figure 5K). From five DAG onward, GUS activity was mainly restricted to hypocotyls and the root regions where root hairs grew but absent in root tips (Figure 5L,M). At seven DAG, seedlings began generating true leaves, from which GUS activity was absent but slightly increased in main roots rather than axillary roots and root tips (Figure 5M). These results indicated that the *HbMFT1* promoter activity was mainly limited to roots, the base of axillary meristems, mature seeds, hypocotyls of post-germinated seedlings and stamens of flowers in transgenic plants, not completely consistent with the expression pattern in rubber tree, in which *HbMFT1* showed predominant expression in seeds and male flowers.

### 2.6. Activity Analysis of GUS Fused with HbMFT1 Promoter in Response to ABA Treatment

**Figure 6:**
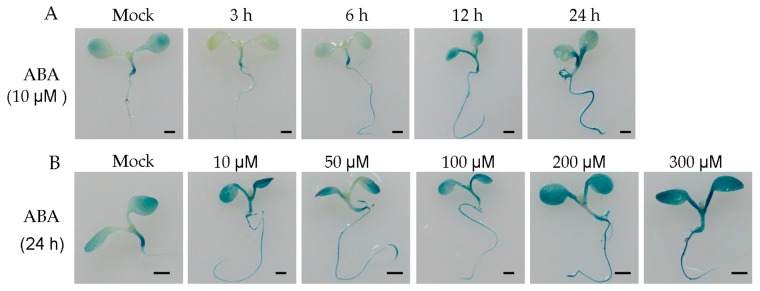

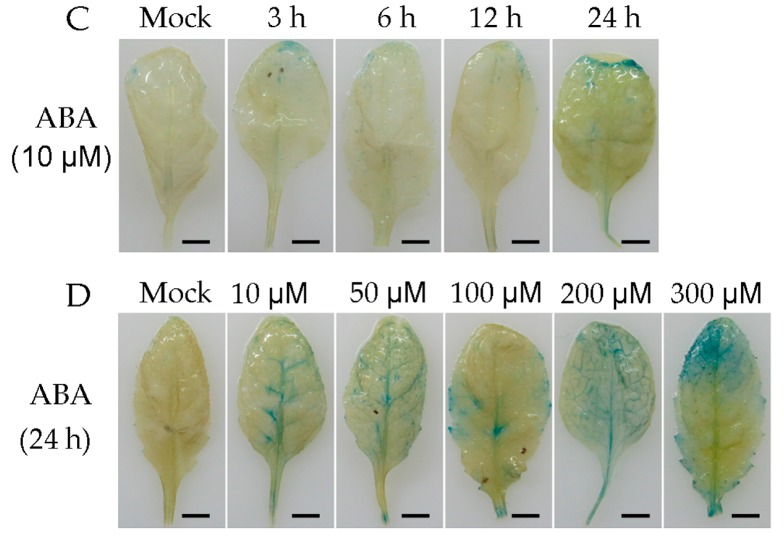
Effect of ABA on HbMFT1::GUS activity in transgenic *Arabidopsis*. (**A**,**C**) The phenotypes of seedlings and rosette leaves of transgenic *Arabidopsis* treated with 10 μM ABA for 3, 6, 12 and 24 h, respectively; (**B**,**D**) The phenotypes of seedlings and rosette leaves of transgenic *Arabidopsis* treated with different concentrations of ABA (10, 50, 100, 200 and 300 μM) for 24 h. Both of the transgenic *Arabidopsis* plant lines with *HbMFT1::GUS* fusions were sampled independently twice. Bar = 1 mm for (**A**,**B**); bar = 3 mm for (**C**,**D**).

*MFT* homologs are regulated by ABA in some species [22,30] and five ABA *cis*-elements were found in *HbMFT1* promoter in this study (Table S1). Therefore, we supposed that *HbMFT1* promoter also responded to ABA. In order to confirm our hypothesis, we treated seven-day-old seedlings and rosette leaves of two transgenic lines, respectively, with 10 μM ABA [22] for 3, 6, 12 and 24 h, respectively. The treatment analysis demonstrated that the HbMFT1::GUS activity of the two lines was notably induced at 24th h and that the two lines had more strong blue stains distributed in cotyledons and roots of seedlings and the sides of rosette leaves than the control (Figure 6A,C). Subsequently, ABA treatment at 10, 50, 100, 200 and 300 μM for 24 h, was used. The results indicated that all concentrations of ABA could induce GUS activity at 24th h, and that 200 and 300 μM of ABA induced the highest GUS activity in seedlings and rosette leaves (Figure 6B,D). However, the transgenic seedlings and rosette leaves without ABA treatment retained the background GUS activity (Figure 6B,D). Therefore, combining the fact of ABA *cis*-elements present in the *HbMFT1* promoter and the result from the effect of ABA on activity of *HbMFT1::GUS* fusion in transgenic plants, it was reasonable to confirm that ABA acts as an activity inducer of the *HbMFT1* promoter.

### 2.7. Overexpression of HbMFT1 Inhibited Seed Germination and Seedling Growth in Transgenic Arabidopsis Plants

**Figure 7:**
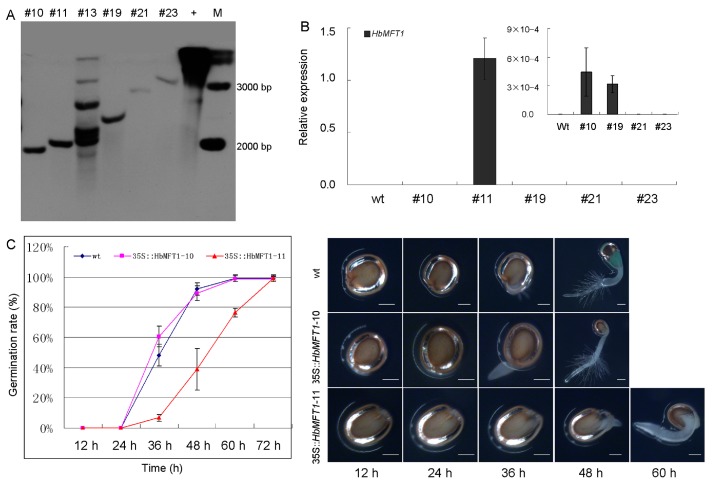
Seed germination comparison between 35S::*HbMFT1* transgenic *Arabidopsis* and wild-type (wt). (**A**) Southern blot analysis of transgene integration of *HbMFT1*; (**B**) Expression analysis of *HbMFT1* in lines transformed with 35S::*HbMFT1*. Values were means ± SE from three independent biological replicates; (**C**) Time course of the germination rate after transformation with 35S::*HbMFT1*; (**D**) Seed germination observation at different developmental periods. Results came from six independent biological replicates. Bar indicates 0.2 mm.

**Figure 8:**
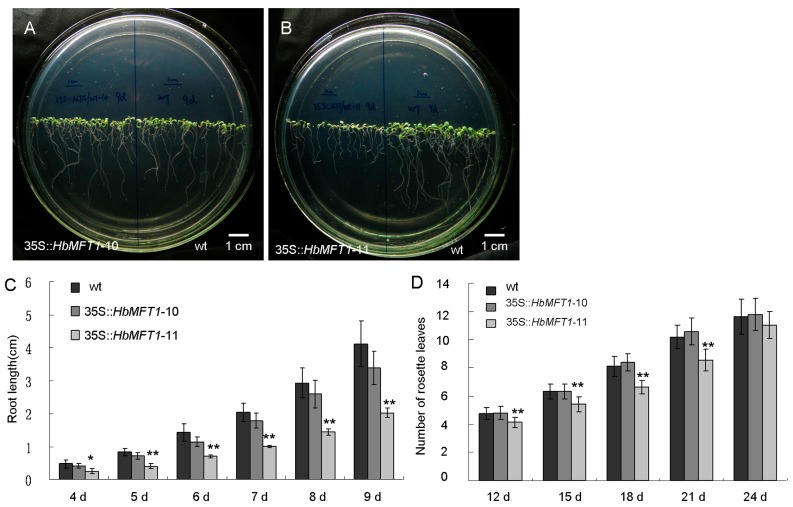
The effect of over-expressing *HbMFT1* on aerial parts and roots growth. (**A**,**B**) Root growth observation for wt and 35S::*HbMFT1* transgenic lines; (**C**) Comparison of root length between wt and 35S::*HbMFT1* transgenic lines; (**D**) Comparison of rosette leaves between wt and 35S::*HbMFT1* transgenic lines. Significant difference tests were carried out using the Student’s *t*-test between wt and 35S::*HbMFT1* transgenic lines. The levels of significance: * indicates 0.01 < *p* < 0.05; ** refers to *p* < 0.01.

*HbMFT1* had the highest expression in embryos, and we speculated that it mainly functions to regulate seed development. To support this hypothesis, we compared the germination rate between the 35S::*HbMFT1* transgenic and the wild-type (wt) *Arabidopsis* plants. More than 50 independent 35S::*HbMFT1* transgenic *Arabidopsis* lines were obtained in the first generation. Through southern blot, we obtained five independent homozygous lines in the third generation (Figure 7A). The qRT-PCR result showed that the expression of *HbMFT1* was detected in line 35S::*HbMFT1*-10, 11 and 19, especially 35S::*HbMFT1*-11, in which the expression was the highest and remarkably higher than that of 35S::*HbMFT1*-10 and 19 (Figure 7B). Therefore, we chose 35S::*HbMFT1*-10 and 11 for further function analysis. A notable observation was that during germination, 35S::*HbMFT1*-11 exhibited notably delayed germination as compared to 35S::*HbMFT1*-10 and wt plants (Figure 7C). After vernalization for three days, wt and 35S::*HbMFT1*-10 *Arabidopsis* showed a respective germination rate of 48.34% and 60.55% at 36 h after germination (HAG), whereas 35S::*HbMFT1*-11 showed a germination rate of only 6.89% (Figure 7C,D). Up to 48 HAG, the germination rates for wt and 35S::*HbMFT1*-10 were 92.06% and 89.21%, respectively, but still very low for 35S::*HbMFT1*-11 (38.87%). Finally, wt and 35S::*HbMFT1*-10 *Arabidopsis* completely germinated at 60 HAG, and 35S::*HbMFT1*-11 at 72 HAG. Intriguingly, at 12 HAG, both wt and 35S::*HbMFT1*-10 yielded distinctly bushy and vigorous root hairs, which were barely observed in 35S::*HbMFT1/*-11 (Figure 8D). In order to further confirm the reliability of this experiment, we chose some other lines from 50 lines of T2 generation to repeat the study of seed germination (Figure S1). The results showed that lines 35S::*HbMFT1*-20, 38 and 41 exhibit a delay in germination similar to that of the 35S::*HbMFT1*-11 line with high expression of *HbMFT1*, although they were not reflected in the southern blot and expression analysis of *HbMFT1*. Moreover, the root growth of 35S::*HbMFT1*-11 was also severely inhibited, whereas 35S::*HbMFT1*-10 exhibited a slight suppression but was not significantly influenced as compared to wt (Figure 8A,B). At four days after germination (DAG), the roots of wt, 35S::*HbMFT1*-10 and 11 grew 4.8, 4.1 and 2.5 mm long, respectively, and then increased to 41.2, 33.9 and 20.3 mm long, respectively, by nine DAG (Figure 8C). For aerial parts, 35S::*HbMFT1*-11 grew more slowly, producing significantly less rosette leaves than wt and 35S::*HbMFT1*-10 (Figure 8D). These results indicate that *HbMFT1* negatively controls seed germination, growth and development.

### 2.8. Overexpression of HbMFT1 Delayed Flowering Time in 35S::HbMFT1 Transgenic Arabidopsis Plants

**Figure 9:**
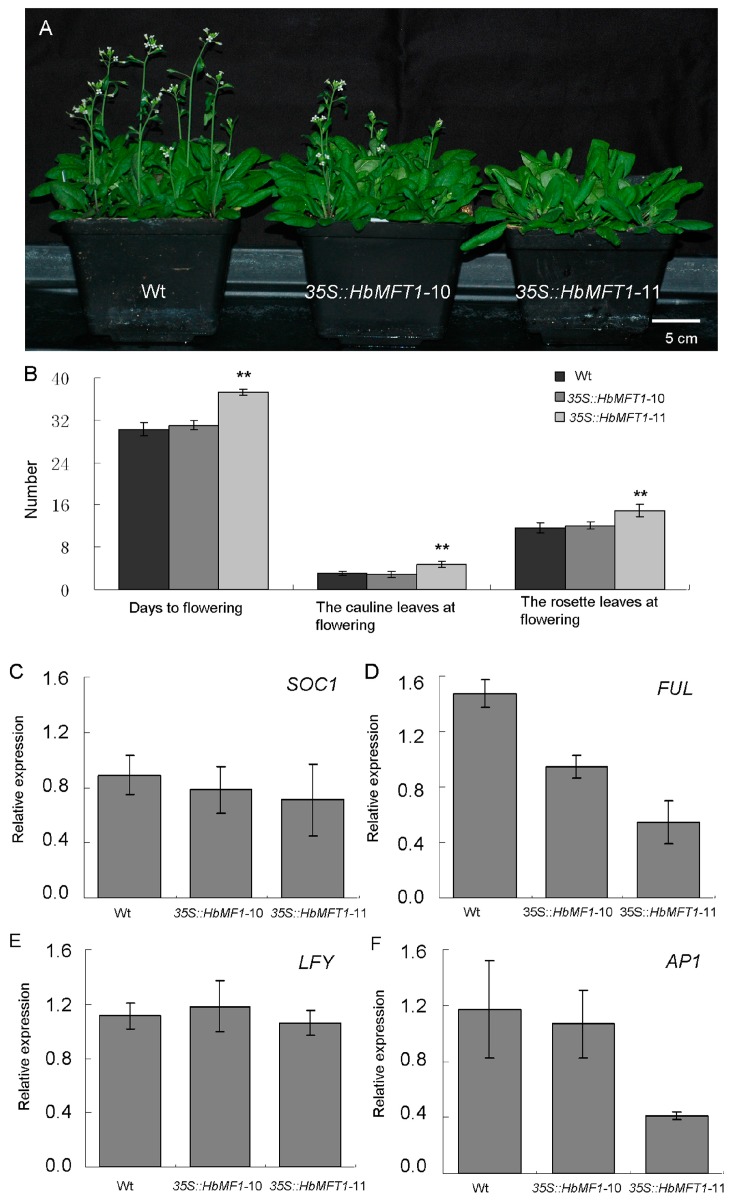

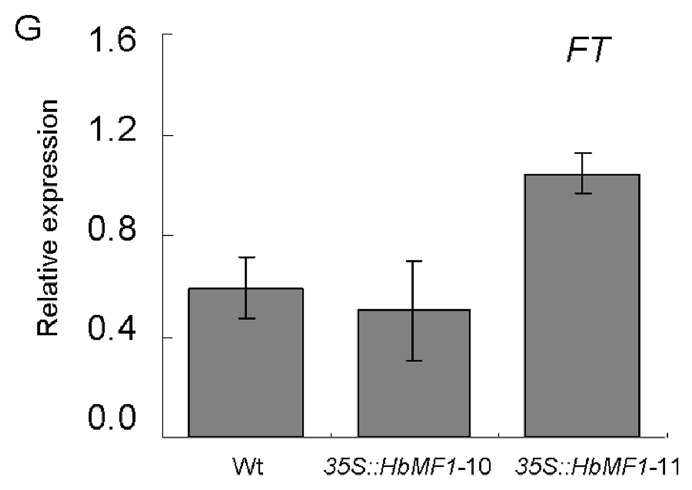
Effect of over-expressing *HbMFT1* on flowering. (**A**) Flowering phenotypes of wt and 35S::*HbMFT1* transgenic lines; (**B**) Comparison of flowering time and number of cauline and rosette leaves at flowering between wt and 35S::*HbMFT1* transgenic lines; (**C**–**G**) Expression analysis of genes related with flowering in wt and 35S::*HbMFT1* transgenic lines. Total RNA was isolated from seedlings growing at 25 days after germination, which was the transition phase of *Arabidopsis* bolting. Values were means ± SE from three independent biological replicates. The levels of significance: ** refers to *p* < 0.01. Abbreviations: *SOC1*, *SUPPRESSOR OF OVEREXPRESSION OF CONSTANS 1*; *LFY*, *LEAFY*; *FUL*, *FRUITFULL*; *AP1*, *APETALA1*; *FT*, *FLOWERING LOCUS T*.

In order to determine whether expression of *HbMFT1* could affect plant architecture, we analyzed the phenotype of 35S::*HbMFT1* transgenic plants. Through morphological observation, overexpression of *HbMFT1* did not cause any obvious morphological change in *Arabidopsis* under long-day conditions but delayed floral initiation. As shown in Figure 9A, 35S::*HbMFT1*-11 flowered at 37.24 ± 0.84 DAG, while 35S::*HbMFT1*-10 and wt flowered at 31.09 ± 0.83 and 30.32 ± 1.25 DAG, respectively. In addition, 35S::*HbMFT1*-11 produced 4.76 ± 0.60 cauline leaves and 14.94 ± 1.15 rosette leaves, significantly higher than those of wt (3.05 ± 0.40 cauline leaves and 11.63 ± 0.96 rosette leaves). As expected, 35S::*HbMFT1*-10 produced similar numbers of cauline and rosette leaves to wt due to its faint accumulation of *HbMFT1* (Figure 9B). Further analysis indicated that the delayed flowering time was related with higher expression levels of *HbMFT1* and down-regulation of *SUPPRESSOR OF OVEREXPRESSION OF CONSTANS 1* (*SOC1*), *LEAFY* (*LFY*), *APETALA1* (*AP1*) and *FRUITFULL* (*FUL*) in transgenic plants, especially the expression levels of *AP1* and *FUL*, which were notably reduced (Figure 9C–F), even though the expression of *AtFT* in 35S::*HbMFT1*-11 was relatively higher than that of wt and 35S::*HbMFT1*-10 (Figure 9G).

## 3. Discussion

Unlike *FT-/TFL-like* genes determined to be “florigen” and “antiflorigen”, respectively, as a member of PEBP family, *MFT-like* genes were not intensively studied in most plant species, and their functions are hence less documented. In the present study, the expression of *HbMFT1* in embryos (Figure 3A) was in agreement with the high HbMFT1::GUS fusion activity in mature seeds of transgenic *Arabidopsis* (Figure 5E) and the seed-specific *cis*-elements found in *HbMFT1* promoter (Table S1), confirming that the primary activities of *HbMFT1* in seed or embryo were similar to those of *MFT* homologs in *Arabidopsi* [22], wheat [25], *Jatropha* *curcas* [30], *Picea* *abies* [23], *Zea mays* [31], *Citrus unshiu* [32], rice [33] and tomato [34]. However, in *orchid*, abundance of the *DnMFT* transcript was strongly detected in auxiliary buds and leaves [35]. Overexpression of *HbMFT1* gave rise to significant delay in seed germination in transgenic *Arabidopsis* relative to wild-type plants, as described for wheat (*Triticum* *aestivum*), in which *Ta-MFT* was induced by low temperature in mature seeds and further confirmed to inhibit germination as expressed in immature embryos driven by the maize (*Zea mays*) ubiquitin promoter [25]. Therefore, transgenic results in the study revealed that *HbMFT1* plays a critical role in maintenance of seed development and maturation. However, *AtMFT* in *Arabidopsis* directly inhibited *ABI5* through a negative feedback mechanism in response to ABA, thereby attenuating the effect of ABA on the suppression of germination [22], functioning analogously to *HbMFT1* and *Ta-MFT*.

In addition, the expression level of *HbMFT1* was progressively increased as inflorescence developed, suggesting that *HbMFT1* may also be involved in the development of reproductive organs. In transgenic *Arabidopsis* plants ectopically expressing *HbMFT1::GUS* fusion, the stamens exhibited strong HbMFT1::GUS activity, which was weakly detected in sepals and petals and was consistent with pollen-specific *cis*-element existing in the promoter (Table S1), indicating that the *HbMFT1* promoter conferred GUS activity preferential expression in stamen and may function to regulate development of the stamen. This might explain why the expression level of *HbMFT1* was almost two-fold more abundant in the male flowers than the female flowers in rubber tree (Figure 3A). *MFT* homologs were also speculated to be associated with development of reproductive organs in other plant species. Four *MFT-like* genes in *Physcomitrella patens* exhibited high expression in gametangia, and *PpMFT2* and *PpMFT4* showed strong expression in the sporophyte, suggesting their association with development of reproductive organs [26]. A high expression of *PaMFT1* in *Picea* *abies* was also detected in pollen [23].

In transgenic *Arabidopsis*, high expression levels of *HbMFT1* may affect development in two aspects. One was that post-embryo growth and development of transgenic *Arabidopsis* was dramatically repressed, with lower germination, shorter roots and fewer rosette leaves compared to wt before floral transition (Figure 7 and Figure 8). The other one was that flowering was delayed, with more rosette and cauline leaves and down-regulation of *SOC1*, *LFY*, *AP1* and *FUL* (Figure 9), each of which is the down-stream gene of *FT* and assigns a floral fate to meristem [11,36,37,38,39,40,41], especially the transcript accumulations of *AP1* and *FUL* that were reduced significantly, whereas the expression of *LFY* similar to *SOC1* was not affected notably due to the fact that *SOC1* is the direct upstream transcriptional activator of *LFY* [42,43]. *AP1*, known as a key floral meristem identity gene, is required to specify the identity of floral meristem in *Arabidopsis* [44]. Furthermore, *FUL* contributed to advancing floral initiation in addition to silique development [45]. Therefore, in the present study, *HbMFT1* may negatively control the activity of *AP1* and *FUL* through a kind of signalling pathway, thereby delaying formation of flowers. *HbMFT1* as a development and flowering inhibitor acted similarly to *MFT* homologs reported in other plant species, such as orchid (*Dendrobium* *nobileLindl*), white and sitka spruce (*P. glauca* and *P. sitchensis*, respectively), of which each *MFT* homolog resulted in relative delay in flowering as heterologously expressed in *Arabidopsis* [35,46]. However, in grapevine, *VvMFT* transcript was mainly detected in shoot and the expression pattern was related with determination of inflorescence meristem as a flowering promoter [47], even though it was more closely related to *HbMFT1* in genetic relationship. In *Arabidopsis*, Yoo *et al.* [21] found that over-expression of *MFT* in wild-type *Arabidopsis* could slightly accelerate flowering, but no remarkably delayed flowering phenotype was found in *mft-1* mutant, suggesting its partial functional redundancy with *FT*. Overall, these results suggested that *MFT* homologs may exert different functions among plant species and their differences have yet to be investigated.

ABRE elements, to date, have been widely studied and confirmed to be involved in controlling the *MFT* promoter activity during seed development. In *Arabidopsis*, ABA-INSENSITIVE3 (ABI3) and ABI5 were considered to be two important ABA signaling components, both of which could directly bind to the *MFT* promoter through recognizing ABRE element to regulate germination of seeds in the ABA-signaling pathway [22]. ABI5 acted as a direct transcriptional promoter of *MFT*, which in turn inhibited the expression of *ABI5*, yielding a negative feedback loop, whereas ABI3 acted as a direct transcriptional repressor of *MFT*. *ABI3* was identified to be an upstream promoter of *ABI5* [48]. During seed development of *Jatropha* and *Picea* *abies*, similar expression patterns between *ABI3* and *MFT* homologs were observed [23,30], suggesting that ABI3 indirectly promotes *MFT* transcriptional level by anegative feedback loop, so that *MFT* maintains seed maturation. Additionally, it has been reported that seed- or embryo-specific expression regulation depends on an ABA-responsive complex [32]. Therefore, in the present study, the highest expression of *HbMFT1* in seeds of rubber tree suggest that it may regulate seed development depending on the ABA-signalling pathway in that the *HbMFT1* promoter contains several ABRE elements (Table S1) and was confirmed to increase GUS activity in transgenic *Arabidopsis* with *HbMFT1::GUS* fusion when exposed to exogenous *ABA* stress (Figure 6).

*HbMFT1* was mainly induced in short-day conditions and showed small oscillations in darkness (Figure 4A), suggesting that expression of *HbMFT1* is regulated through both photoperiod and the circadian clock. Photoperiodic expression of the *PEBP* family has been reported in other species. Four homologs of *MFT* in *Physcomitrella patens* showed a peak expression after approximately 2 h after the onset of light in long days [26]. In addition, *AtBFT* [19], *AtFT* [19] and *DnFT* [35] were induced under long-day conditions. On the contrary, *Hd3a* [34] and *ATC* [20] were induced under SD conditions and showed a rhythmic expression.

In conclusion, we characterized *HbMFT1* gene as a multifunctional regulator in rubber tree based on tissue-specific expression, temporal and spatial expression, putative plant regulatory elements analysis and ectopical expression analysis in *Arabidopsis* plants. Transgenic *Arabidopsis* plants ectopically over-expressing *HbMFT1* substantially retarded seed germination, growth and development and flowering. Abundant HbMFT1::GUS activities in stamens and mature seeds of transgenic *Arabidopsis* plants were consistent with its expression in rubber tree and the putative seed- and pollen-specific *cis*-elements existing in the promoter. In addition, exogenous ABA could dramatically promote GUS activity in transgenic plants transformed with *HbMFT1::GUS* fusion because of the presence of ABRE elements in *HbMFT1* promoter. Our study suggests that *HbMFT1* may mainly retain the state of seeds maturation and regulate development of stamens in rubber tree. Moreover, seed- and pollen-preferential expressions indicate that the *HbMFT1* promoter is ideal to regulate target gene expression in seeds or stamens of rubber tree or other plant species.

## 4. Materials and Methods

### 4.1. Plant Materials and Growth Conditions

Rubber trees of *Hevea* *brasiliensis* clone CATAS 7-33-97, including 3-month-old seedlings, 2-year-old, and 10-year-old rubber trees, were grown at the experimental plantation of the Rubber Research Institute, Chinese Academy of Tropical Agricultural Sciences (RRI-CATAS), Danzhou, Hainan, China. In order to study the tissue-specific expression of target gene, embryos and roots, stems, shoot apices and four developmental-stage leaves (bronze, color-change, pale-green and mature leaves) of 3-month-old seedlings, five developmental-stage inflorescences (I1: <0.5 cm; I2: =2 cm; I3: =4 cm; I4: =8 cm; I5: >8 cm and flowering), open male flowers, open female flowers and pericarps of 10-year-old rubber trees were collected. In addition, roots, stems, mature leaves and shoot apices of rubber trees at different ages were collected separately to study the spatial and temporal expression pattern of a target gene. In order to determine whether the target gene was influenced by photoperiod, 40 three-month-old seedlings with similar morphological characteristics were divided into two groups, with 20 seedlings each group. One group was planted under long-day condition (16-h light/8-h dark) at 28 °C, whereas the other group was grown under short-day condition (8-h light/16-h dark) at 28 °C. After one month, mature leaves were collected at the initiation of light (8 a.m.) and continued every 2 h during the whole day. We also assessed the effect of temperature on expression of the target gene. Under short-day condition (8-h light/16-h dark), 40 three-month-old seedlings with similar morphological characteristics were equally divided into two groups. The first group was transferred into a growth chamber, in which the culture temperatures were set at 28, 32 and 36 °C, respectively, whereas the second group was transferred into another growth chamber, in which culture temperatures were set at 28, 24, 20, 16 and 8 °C, respectively. Each temperature lasted for 3 days, and then was adjusted to next temperature. To avoid the interference of photoperiod and biological clock on expression, we harvested the mature leaves at the same time (10 a.m.).

Wild-type *Arabidopsis thaliana* seeds (Columbia) were vernalized for 3 days at 4 °C, and then grown on sterilized vermiculite containing 1/2× MS medium for 8 days before transplanted to soil. Seedlings were planted in a growth chamber under long-day conditions (16-h light/8-h dark) at 22 °C.

### 4.2. DNA and RNA Extraction

Genomic DNA from leaves of rubber tree was extracted according to Risterucci *et al.* [49]. The total RNA of both rubber tree and *Arabidopsis* was extracted according to the method of Tang *et al.* [50]. The total RNA extracted was then treated using Dnase I (Thermo Scientific, Waltham, Massachusetts, USA) to avoid contamination of genomic DNA. Agarose gel electrophoresis was used to assess the integrality of total RNA or DNA. The purity and concentration of total RNA, which was used for reverse transcription, was measured by NanDrop 2000 spectrophotometer (Thermo Fisher, Waltham, MA, USA) at wavelength of 230, 260 and 280 nm.

### 4.3. Isolation of Full-Length and Putative Promoter of MFT-Like Gene

*JcMFT* and *AtMFT* from *Jatropha* *curcas* and *Arabidopsis* were used as queries, and the local BLAST program was carried out with leaf transcriptome, which was finished by our research group (data unpublished) to search putative *MFT-like* genes. *MFT-like* genes were amplified by a pair of specific primers, *HbMFT1*(ORF)-F and *HbMFT1*(ORF)-R. In order to obtain the full-length cDNA of the *MFT-like* gene, 3′ rapid amplification of cDNA ends (RACE) was conducted. Three adaptor primers named QT, Q0 and Q1 and two gene-specific primers, GSP1 and GSP2, were designed for 3′ RACE. QT was used as primer for reverse transcription. Q0 and GSP1 were used for the first PCR program while Q1 and GSP2 were used for second PCR program. Amplified products were cloned into pMD19-T cloning vector (TaKaRa) and sequenced. A 2267 bp 5′ flanking region utilized as putative promoter was amplified based on the result of the local BLAST program using genome database (unpublished data) and the genomic open reading frame sequence (ORF) of the *MFT-like* gene. The primer sequences used for 3’ UTR, ORF and promoter amplification are shown in Table S2.

### 4.4. Bioinformatic Analysis

Genomic organization of the target gene was analyzed by aligning the cDNA with its corresponding genomic DNA using the online web server Clustal Omega (http://www.ebi.ac.uk/Tools/msa/clustalo/) [51]. Multiple sequence alignment between target proteins and the PEBP family of other species was carried out using DNAMAN software 6.0 version (Lynnon Biosoft, San Ramon, CA, USA). For evolutionary analysis, we used MEGA (Molecular Evolutionary Genetics Analysis) software package version 5.0 (www. megasoftware.net) [52], in which N–J method with ClustalW software was used and N–J tree was produced from the results of 1000 bootstrap replicates [52].

### 4.5. Expression Analysis of Related Gense in Rubber Tree and Arabidopsis Transgenic Lines

We carried out quantitative reverse transcriptase-polymerase chain reaction (qRT-PCR) to assess the expression of target genes. First-strand cDNA was synthesized from three micrograms of total RNA in 20 µL reaction mixtures according to the manufacturer’s instructions (RevertAid™ First Strand cDNA Synthesis Kit, Fermentas, Waltham, MA, USA). The qRT-PCR was conducted in a reaction volume of 10 µL, including 30 ng cDNA per sample, 1× SYBR^®^PremixExTaq^TM^ (TAKARA Biothnology Corporation, Dalian, China) and 0.2 μM each primer, and performed in 384-well plates with the CFX384 system (Bio-Rad Laboratories, Hercules, CA, USA). Relative expression analysis of each gene was calculated by Pfaffl method [53]. *At2g28390* and *At3G01150* from *Arabidopsis* [19,54,55] and *HbRH2b*, *HbRH8* and *HbYLS8* from rubber tree were used as reference genes in qRT-PCR [56]. Related primer sequences and reaction programs used for qRT-PCR are described in Table S3.

### 4.6. Construction of Binary Vector for Target Gene and Promoter-GUS Fusion and Transformation

Target genes were amplified based on a pair of specific primers (shown in Table S2), in which the *EcoR*I and *Xba*I enzyme site sequences were added in the 5′ regions of forward and reverse primers, respectively. The products of amplification were digested with *EcoR*I and *Xba*I and cloned into the *EcoR*I-*Xba*I sites of pXCS vector which harbours a bar gene conferring resistance to herbicide to replace the multiple clone site (MCS). In addition, in order to construct the vector of promoter::GUS fusion, the above-mentioned 2267 bp *HbMFT1* promoter fragment was subcloned into X*ba*I-N*co*I site of pCAMBIA 3301. Both of these two types of constructions were introduced into *Agrobacterium tumefaciens* GV3101 by electroporation. Eventually, the resultant *Agrobacterium* strains were used for transformation of *Arabidopsis* wild-type plants according to the floral-dip method [57]. For selection of transformants, seeds vernalized for 3 days were plated on vermiculite supplemented with 1/2× MS medium solution for 4 days, and then sprayed with herbicide Basta at 50 mg/L every 3 days. After 10 days, bar-resistant transformants were selected and transplanted to soil in the growth chamber. Positive transformants were identified by Southern Blot method [58].

### 4.7. Phenotype Analysis of Transgenic Arabidopsis

In order to determine whether over-expression of *HbMFT1* affects seed germination, the seeds of transgenic lines and wild-type (wt) *Arabidopsis* were vernalized for 3 days at 4 °C in 1/2× MS mediums, and then the seed germination was observed and counted using a stereo microscope (LEICA, Germany) per 12 h till the third day (namely 72 h). As for the measurement of root lengths, we took photos of the roots of the wt and transgenic lines, for at least 20 plants each, every day from the fourth to the ninth day after germination with 1 cm as a scale bar. Finally, the actual length of root for each line was calculated using Image-Pro Plus (IPP) software 6.0 (IPP 6.0-Media Cybernetics, Bethesda, MD, USA) based on scale.

### 4.8. ABA Treatment

Mature *Arabidopsis* seeds of transgenic plants were sterilized with solution containing 75% (*v*/*v*) ethanol and 0.05% Triton X-100 for 5 min, and the solution was then removed. These seeds were washed again using 95% ethanol for 5 s and transferred on a sterilized filter paper. When ethanol was completely volatilized, these seeds were sown in the plates with 1/2×MS medium. After vernalization at 4 °C for 3 days, these plates were transferred into a plant growth chamber at 22 ± 2 °C under a 16/8 h (light/dark) condition. At 7 days after germination, seedlings were divided into 10 groups (each group contained at least 6 seedlings), of which the first 5 groups were grown in 1/2× MS medium supplemented with 10 μM ABA for 3, 6, 12 and 24 h, respectively. The rest of the 5 groups of seedlings were grown in 1/2× MS medium supplemented with different concentrations of ABA (10, 50, 100, 200 and 300 μM) for 24 h. 1/2× MS Finally, all of seedlings sampled were stained using GUS solution. With regard to the treatment of rosette leaves with ABA, two independent single plants of each line were randomly selected as samples. Rosette leaves from a single seedling growing at 25 days after germination were treated with ABA at different concentrations and time points, with the same procedure as that for seedlings treatment with ABA. Seedlings and rosette leaves with no ABA treatment were used as controls.

### 4.9. Histochemical GUS Staining

Histochemical GUS analysis was carried out using various tissues and organs in the GUS staining solution at 37 °C overnight, which includes 0.5 mM K_3_Fe(CN)_6_, 0.5 mM K_4_Fe(CN)_6_·3H_2_O, 10% (*v*/*v*) MeOH, 64 mM Na_2_HPO_4_, 32 mM KH_2_PO_4_, 10 mM Na_2_EDTA (pH 8.0), 0.1% (*v*/*v*) Triton X-100 and 1 mM X-Gluc. Finally, the tissues and organs sampled were destained by 70% ethanol and used for observation.

## Supplementary Materials

Supplementary materials can be found at http://www.mdpi.com/1422-0067/17/3/247/s1.

## Abbreviations

*AP1*: *APETALA1*
*ATC*: *ARABIDOPSIS THALIANA CENTRORADIALIS*
*BFT*: *BROTHER OF FT AND TFL1*
*CO*: *CONSTANS*
DAG: Day after germination
*FD*: *FLOWERING LOCUS D*
*FUL*: *FRUITFULL*
*FT*: *FLOWERING LOCUS T*
HAG: Hour after germination
LD: Long-day condition
*LFY*: *LEAFY*
MCS: Multiple clone site
*MFT*: *MOTHER OF FT AND TFL1*
*ORF*: Open reading frame sequences
PEBP: Phosphati-dylethanolamine binding protein
RACE: Rapid amplification of cDNA ends
qRT-PCR: Quantitative reverse transcriptase-polymerasechainreaction
SD: Short-day condition
*SOC1*: *SUPPRESSOR OF OVEREXPRESSION OF CONSTANS 1*
*TSF*: *TWIN SISTER OF FT*
*TFL1*: *TERMINAL FLOWER1*
wt: Wild-type
