# Supplementary material for: Identification, Functional Study, and Promoter Analysis of HbMFT1, a Homolog of MFT from Rubber Tree (Hevea brasiliensis)
Source: Int J Mol Sci. 2016 Mar 2;17(3):247. doi: 10.3390/ijms17030247 (PMC4813128; doi:10.3390/ijms17030247)
Supplement: Supplementary file 1 [file ijms-17-00247-s001.pdf]

Supplementary Materials: Identification, Functional Study, and Promoter Analysis of *HbMFT1*, a Homolog of *MFT* from Rubber Tree (*Hevea brasiliensis*)

Zhenghong Bi, Xiang Li, Huasun Huang and Yuwei Hua

Table S1. Putatuve *cis*-element of *HbMFT1* promoter analysis with PLACE database.

cis-Acting Element	Element Sequence	Element Number	Function
WBOXATNPR1	TTGAC	1	Salicylic acid (SA)-induced element
WBOXHVISO1	TGACT	3	Sugar-responsive elements
WRKY71OS	TGAC	6	GA-responsive element
WBOXNTERF3	TGACY	4	Wounding-responsive element
GATABOX	GATA	3	Seed-specific element
GARE1OSREP1	TAACAGA	1	GA-responsive element
DOFCOREZM	AAAG	17	Endosperm-specific element
SEF4MOTIFGM7S	RTTTTTR	3	Embryo expression element
CCAATBOX1	CCAAT	2	Heat-responsive element
MYBCORE	CNGTTR	3	Water stress-responsive element
NODCON2GM	CTCTT	14	Nodules-expression element
SORLIP1AT	GCCAC	1	Light-responsive element
GTGANTG10	GTGA	8	Pollen expression element
SEBFCONSSTPR10A	YTGTCWC	2	Auxin-responsive element
BIHD1OS	TGTCA	7	Disease-responsive element
GT1CONSENSUS	GRWAAW	9	Light-responsive element
SURECOREATSULTR11	GAGAC	2	Sulfur-responsive element
TAAAGSTKST1	TAAAG	3	Guard cells expression element
POLLEN1LELAT52	AGAAA	5	Pollen-specific element
CURECORECR	GTAC	2	Oxygen-responsive element
ANAERO1CONSENSUS	AAACAAA	1	Oxygen-responsive element
AACACOREOSGLUB1	AACAAAC	1	Endosperm-specific element
EBOXBNNAPA	CANNTG	16	Storage-protein element
GT1GMSCAM4	GAAAAA	2	Light-responsive element
MYCATERD1	CATGTG	1	Storage-protein element
SITEIIATCYTC	TGGGCY	2	Anther- and meristem-specific element
MYCATRD22	CACATG	1	Storage-protein element
2SSEEDPROTBANAPA	CAAACAC	1	Storage-protein element
CANBNNAPA	CNAACAC	3	Embryo- and endosperm-specific element
PROXBBNNAPA	CAAACACC	1	Embryo- and endosperm-specific element
CIACADIANLELHC	CAANNNNATC	2	Circadian expression element
SORLIP2AT	GGGCC	2	Light-responsive element
PYRIMIDINEBOXOSRAMY1A	CCTTTT	2	Sugar-responsive element
NODCON1GM	AAAGAT	2	Nodule-specific element
RAV1AAT	CAACA	2	Rosette leaves- and roots-specific element
DPBFCOREDCDC3	ACACNNG	3	Embryo-specific element
ASF1MOTIFCAMV	TGACG	1	Auxin-and salicylic acid-responsive element
CACGCAATGMGH3	CACGCAAT	1	Auxin-responsive element
SEF3MOTIFGM	AACCCA	1	Embryo-specific element
LTRECOREATCOR15	CCGAC	1	Low temperature-responsive element
CACGTGMOTIF	CACGTG	1	light-responsive element
ABRERATCAL	MACGYGB	2	light-responsive element
ACGTABREMOTIFAOSOSEM	TACGTGTC	2	ABA-responsive element
ABRE3HVA1	GCAACGTGTC	1	ABA-responsive element
QARBNEXTA	AACGTGT	1	JA-responsive element
T/GBOXATPIN2	AACGTG	1	JA-responsive element
ACGTABREMOTIFA2OSEM	ACGTGKC	2	ABA-responsive element
GADOWNAT	ACGTGTC	2	GA-responsive element
TGTCACACMCUCUMISIN	TGTCACA	1	Fruit-specific element
RHERPATEXPA7	KCACGW	1	Root hair-specific *cis*-elements
RAV1BAT	CACCTG	1	Rosette leaves- and roots-specific element

Table S2. The sequences of primers used for amplification of *HbMFT1* gene and its promoter and construction of related binary vectors.

Name	Sequence(from 5′ to 3′)	Feature
Q0	CCAGTGAGCAGAGTGACG	Adaptor primer for 3′ RACE
Q1	GAGGACTCGAGCTCAAGC	Adaptor primer for 3′ RACE
QT	CCAGTGAGCAGAGTGACGAGGACTCGAGCTC<br>AAGCTTTTTTTTTTTTTTT	Used as reverse transcription primer for 3′ RACE
GSP1	AACAAATCGCTAATGGCTGGGAAAT	*HbMFT1* gene-specific primer for 3′ RACE
GSP2	GGCTTCCAGTTGCTGCTGTGTATTTTAACTCG	*HbMFT1* gene-specific primer for 3′ RACE
HbMFT(ORF)-F	GGAATTCCATGGCTCGGTCCCT	Used for cloning the ORF of *HbMFT1* and construction of *35S*::*HbMFT1*.<br>The added E*co*RI was underlined.
HbMFT(ORF)-R	GCTCTAGAGCTCAACGTTTTTTAACAG	Used for cloning the ORF of *HbMFT1* and construction of *35S*::*HbMFT1*.<br>The added X*ba*I was underlined.
*HbMFT*QI-F	GCTCTAGAGGGGTGTAATCTTGACTTCTGCGAC	Used for cloning putative promoter of *HbMFT1* and construction of *HbMFT1*::*GUS*.<br>The added X*ba*I was underlined.
*HbMFT*QI-R	CATGCCATGGAGTTATGATCAAACAGGACCCA<br>GGTGGTGCC	Used for cloning putative promoter of *HbMFT1* and construction of *HbMFT1*::*GUS*.<br>The added N*co*I was underlined.

Table S3. Gene-specific primers for qRT-PCR.

Primers(QF/QR) *	Program	Sequence	Primer Efficiency	References
HbYLS8(reference)-QF<br>*HbYLS8*(reference)-QR	C	CCTCGTCGTCATCCGATTC<br>CAGGCACCTCAGTGATGTC	1.98	[56]
HbRH2b(reference)-QF<br>*HbRH2b*(reference)-QR	D	AGGTGGATTGGCTAACTGAG<br>GAGCCCAAACATCAGTAGTG	1.99	[56]
HbRH8(reference)-QF<br>*HbRH8*(reference)-QR	D	TCACAGGGTTGGTAGATCAG<br>CCAAGCTCTTGCTCAATCC	1.99	[56]
HbMFT1-QF [a]<br>*HbMFT1*-QR [a]	B	GTGACCCAAAACAAGAAACAAT<br>ATCAGAGGAGAAACGAGAACC	1.95	–
At2g28390(reference)-QF<br>At2g28390(reference)-QR	B	TTGCAGACAAGGCGATGG<br>GCAAGAACACTTCATTCTCCACA	1.94	[55]
At3G01150(reference)-QF<br>AT3G01150(reference)-QR	C	CGTCATACTGATCTGAATGTTAAGGCTTTTAGCG<br>CATAGTAAGGAGGTTGACCTGAGGGTG	1.95	[19,54]
Target(HbMFT1)-QF [b]<br>*Target(HbMFT1)*-QR [b]	D	GTCCGCAAAAATCACCAGTCTCT<br>CCCTTATCTGGGAACTACTCACAC		
AtAP1-QF<br>*AtAP1*-QR	A	CTCCATAAATAAAGATCCCGAGACTCAA<br>CTAAAAGAACCAAACAAAACAAAGACCC	1.98	
AtLFY-QF<br>*AtLFY*-QR	C	TGCTAAAGACCGTGGCGAAA<br>AACAAGCCTGACGCCATGAG	1.96	
AtFUL-QF<br>*AtFUL*-QR	D	TTGCAAGATCACAACAATTCGCTTCT<br>GAGAGTTTGGTTCCGTCAACGACGAT	1.94	
AtSOC1-QF<br>*AtSOC1*-QR	C	ACACAAATAGATGAAACGAGGAAAG<br>TGTATGTATAGGTAGAGATATAGGAGGAGACT	1.96	

A 95 °C for 30 s; 40× (95 °C for 10 s , 56 °C for 20 s, and 72 °C for 20 s); B 95 °C for 30 s; 40× (95 °C for 10 s , 60 °C for 20 s, and 72 °C for 20 s); C 95 °C for 30 s; 40× (95 °C for 10 s , 62 °C for 20 s, and 72 °C for 20 s); D 95 °C for 30 s; 40× (95 °C for 10 s, 64 °C for 20 s, and 72 °C for 20 s); * QF and QR represent forward and reverse primers for qRT-PCR, respectively; [a] represents forward and reverse primers of *HbMFT1* gene used for qRT-PCR in rubber tree; [b] represents forward and reverse primers of *HbMFT1* gene used for qRT-PCR in *Arabidopsis* plant.

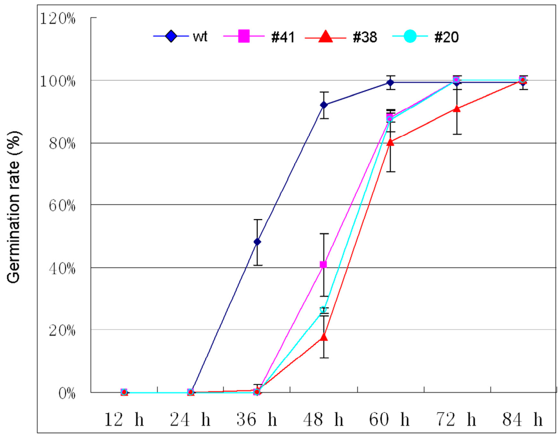

Figure S1. Time course of the germination rate after transformation with 35S::*HbMFT1*.
